# Combination of Pioglitazone and Metformin Actions on Liver Lipid Metabolism in Obese Mice

**DOI:** 10.3390/biom13081199

**Published:** 2023-07-31

**Authors:** Jieying Liu, Dongmei Wang, Ziyan Xie, Lu Ding, Shunhua Li, Xuemei Ma, Jing Liu, Jing Ren, Cheng Xiao, Chunru Yang, Xinhua Xiao

**Affiliations:** 1 Department of Endocrinology, NHC Key Laboratory of Endocrinology, Peking Union Medical College Hospital, Peking Union Medical College, Chinese Academy of Medical Sciences, Beijing 100730, China; liujieying50@pumch.cn (J.L.); wdongmay@126.com (D.W.);; 2 Department of Medical Research Center, Peking Union Medical College Hospital, Peking Union Medical College, Chinese Academy of Medical Sciences, Beijing 100730, China

**Keywords:** metformin, pioglitazone, combination therapy, NAFLD, lipidomics analysis

## Abstract

Background: Despite the increasing prevalence rate of nonalcoholic fatty liver disease (NAFLD) worldwide, efficient pharmacotherapeutic regimens against NAFLD still need to be explored. Previous studies found that pioglitazone and metformin therapy could partly ameliorate NAFLD, but their combination therapy effects have not been researched. In the present study, we assessed the protective effects of metformin and pioglitazone combination therapy on liver lipid metabolism in high-fat diet (HFD)-fed mice and investigated the molecular mechanism. Methods: Male C57BL/6 mice were divided into five groups: normal control; HFD control; metformin monotherapy; pioglitazone monotherapy and combined therapy. After 8 weeks of pharmacological intervention, glucose and lipid metabolism characteristics, hepatic histology, lipidomics profiling and RNA-seq analysis were performed. Results: The combination of pioglitazone and metformin significantly ameliorated HFD-induced metabolic disturbance and the hepatic oil red O area. A lipidomics analysis showed that combined therapy could significantly reduce the high levels of free fatty acids (FFA), diacylglycerol and triglycerides, while a set of glycerophospholipids and sphingolipids were increased in the combined therapy group. Consistently, an RNA-seq analysis also showed a remarkable reduction in genes associated with FFA uptake and de novo lipogenesis, including *Cd36*, *Fads1*, *Fads2*, *Fasn*, *Scd1*, *Elovl5* and *Pklr* in the combined therapy group. Conclusions: Pioglitazone and metformin might have a synergistic protective effect on NAFLD by improving hepatic lipid profiles in HFD-induced mice. Further studies are needed to verify the clinical effects.

## 1. Introduction

Previous studies have reported that the worldwide population prevalence and incidence of nonalcoholic fatty liver disease (NAFLD) is approximately 25% [1]. Characterized by chronic excessive intrahepatic fat deposition (steatosis) without excessive alcohol use, NAFLD, also termed metabolic-associated fatty liver disease, has a strong relationship with obesity, type 2 diabetes mellitus (T2DM) and cardiovascular disorders [2]. Healthy lifestyle management, such as physical activity, dietary patterns and healthy habits, does not sustainably reduce liver steatosis in most patients [3,4]. Without appropriate intervention, a fatty liver is highly likely to damage a patient’s health condition, which can lead to cirrhosis and liver cancer [3]. However, there are currently no approved pharmacological treatment regimens for NAFLD [4].

A vast body of research has found that insulin resistance (IR) is a major evolutionary force that can result in excessive lipid accumulation in hepatocytes and the subsequent progression of hepatitis and fibrosis [5]. Thus, various antidiabetic agents have been shown to be efficient hepatic steatosis therapeutics in animal models or clinical research [5,6,7]. As typical insulin sensitizers, thiazolidinediones (TZDs), are peroxisome proliferator-activated receptor γ (PPARγ) agonists that might alleviate hepatic steatosis by boosting triglyceride (TAG) storage in adipocytes, thereby transferring the lipid burden towards adipose tissue rather than the liver and leading to decreased circulating free fatty acids (FFA) levels and hepatic lipid accumulation [4,8]. A meta-analysis showed that pioglitazone significantly improved liver ballooning degeneration, lobular inflammation and fibrosis in patients with nonalcoholic hepatitis [9]. Therefore, TZDs have been evaluated as possible pharmacotherapies for patients with NAFLD in clinical care [10,11]. In addition to TZDs, metformin is also considered an insulin-sensitizing agent [5,12]. A large number of experimental studies have found that metformin has a significant effect on NAFLD in mouse, rat and zebrafish models [13,14,15,16]. The underlying mechanism might include reducing de novo lipogenesis, promoting free fatty acids (FFA) oxidation and modulating immune inflammation [13,14,15,16]. In clinical studies, metformin was proven to reduce ALT levels in pediatric NAFLD and was associated with a lower risk of hepatocellular carcinoma [7,17]. However, the results were inconsistent in other clinical studies [18,19]. Two previous meta-analyses suggested that metformin could not alleviate liver histology in NAFLD and steatohepatitis [20,21].

Due to the progressive nature of T2DM, glycemic control with monotherapy is difficult to achieve for most diabetes patients [22,23]. Therefore, the above-mentioned insulin sensitizers, that is, fixed-dose pioglitazone/metformin tablets, are approved for the treatment of T2DM patients who have inadequate maintenance of glucose homeostasis with monotherapy [23]. In our previous study, we found that the combined effect of metformin and pioglitazone significantly improved glucolipid metabolic parameters as well as modified gut microbiota and metabolites compared with monotherapy in high-fat diet (HFD)-induced obese mice [24]. Based on previous evidence, we hypothesized that metformin and pioglitazone combined therapy would play a synergetic role in the improvement of liver steatosis in HFD-fed mice. In the present study, we assessed the effects of metformin and pioglitazone combination therapy mainly on liver lipid metabolism in HFD-fed mice.

## 2. Materials and Methods

### 2.1. Animals and Study Design

The experimental animal models were described in our previous study and we repeated the experimental protocol in this study [24]. Seven-week-old male C57BL/6 mice were obtained from Beijing Vital River Laboratory Animal Technology Co., Ltd. (Beijing, China, SCXK-2021-0011). All mice were raised in a standard specific pathogen-free environment (22 ± 2 °C under a 12 h light/12 h dark cycle). During the experimental period, all the mice had free access to food and water. After one week of acclimatization, a total of 30 mice were randomly divided into five groups (n = 6 per group): the control diet group (Ctr), HFD control group (HFD), HFD with metformin intervention group (HFD + Met), HFD with pioglitazone intervention group (HFD + Pio) and HFD with metformin and pioglitazone combination intervention group (HFD + Met + Pio). Mice were raised on a normal control diet (AIN-93G) or HFD (D12492) during the first ten weeks. The HFD diet included 60% of calories from fat, whereas the normal control diet contained 15.8% of calories derived from fat. The type of fat in HFD included soybean oil and lard, without cholesterol. The calorific value of the HFD diet and normal control diet were 5.24 and 3.9 kcal/g, respectively. Then, different pioglitazone or metformin interventions were given for the last eight weeks: the HFD + Met group was continued on the HFD and received metformin (150 mg/kg/day body weight), the HFD + Pio group was continued on the HFD and received pioglitazone (5 mg/kg/day body weight) and the HFD + Met + Pio group was continued on the high-fat diet and treated with metformin (150 mg/kg/day body weight) combined with pioglitazone (5 mg/kg/day body weight). The Ctr and HFD groups received vehicle (0.5% carboxymethyl cellulose sodium) for 8 weeks by intraperitoneal injection in a similar manner to the other three groups receiving drug treatment. Metformin and pioglitazone were purchased from Hangzhou Zhongmei Huadong Pharmaceutical Co., Ltd., Hangzhou, China. At the end of the experiment, all mice were sacrificed and serum samples and tissues were collected for further analyses. The animal care and use committee of the Peking Union Medical College Hospital approved all of the procedures (Beijing, China, XHDW-2022-028). All animal procedures in our experiments were performed in strict accordance with the National Institutes of Health Guide for the Care and Use of Laboratory Animals.

### 2.2. Glucose and Insulin Tolerance Tests

The oral glucose tolerance test (OGTT) and insulin tolerance test (ITT) were performed at the end of the intervention by gavage. For the OGTT, mice fasting for 14 h were orally administered a glucose load (2 g/kg body weight). For the ITT, mice fasting for 6 h were injected intraperitoneally with short-acting insulin (1.0 U/kg bodyweight). The glucose levels were evaluated using a glucometer (FreeStyle Optium™, Abbott, Chicago, IL, USA) after blood samples were taken from the tail vein at six separate time periods for OGTT and five separate time periods for ITT (0, 15, 30, 60, 90 or 120 min). The areas under the curve (AUCs) for the OGTT and ITT were calculated as previously described [24].

### 2.3. Biochemical Parameter Measurements

Blood samples were collected from the intraorbital retrobulbar plexus at the end of the intervention after 14 h of fasting and were centrifuged at 3000× *g* for 10 min at 4 °C. An ELISA kit (80-INSMSU-E01, Salem, NH, USA) was used to assess serum insulin levels. Serum glucose, TAG, total cholesterol (TC), FFA, alanine aminotransferase (ALT) and aspartate aminotransferase (AST) were measured using an autoanalyzer at Peking Union Medical College Hospital, as previously described [24]. Insulin sensitivity was calculated using the homeostasis model assessment of insulin resistance (HOMA-IR) [25].

### 2.4. Histology Examination

A lobe of liver samples was fixed in phosphate-buffered 10% formalin and then embedded in paraffin wax for hematoxylin and eosin (HE) staining. At the same time, another lobe of liver samples was embedded in a Tissue-Tek optimum cutting temperature compound for oil red O staining [26]. Oil red O staining was performed using an oil red O staining kit (BASA-4081A, ZSJQ Bio, Beijing, China) according to standard protocols. Quantitative analysis was performed by Image Pro Plus 6.0 software.

### 2.5. Lipidomics Analysis

Frozen liver pieces were prepared for lipidomics analysis. Liquid chromatography-mass spectrometry (LC-MS) analysis was carried out using SCIEX ExionLC series UHPLC System with a UPLC HSS T3 column (2.1 mm × 100 mm, 1.8 μm) coupled with an AB Sciex QTrap 6500+ mass spectrometer. Data preprocessing and annotation were performed using Biobud-v2.0.7 Software. The final dataset containing the information on the compound name, sample name and concentration was imported to the SIMCA software (V16.0.2, Sartorius Stedim Data Analytics AB, Umea, Sweden) package (Sartorius Stedim Data Analytics AB, Umea, Sweden) for multivariate analysis. The data were scaled and logarithmic transformed to minimize the impact of both noise and high variance of the variables. After these transformations, principal component analysis (PCA) was carried out to visualize the distribution and grouping of the samples. The metabolites with variable important in projection (VIP) >1 and *p* < 0.05 (student *t*-test) were considered as significantly changed metabolites.

### 2.6. RNA-seq Analysis

RNA was extracted from frozen livers using TRIzol reagent (Life Technologies Inc., Carlsbad, CA, USA) according to the manufacturer’s instructions. Total amounts and integrity of RNA were assessed using the RNA Nano 6000 Assay Kit of the Bioanalyzer 2100 system (Agilent Technologies, CA, USA). Library preparation for RNA-seq was performed using NEBNext^®^ Ultra™ RNA Library Prep Kit (Illumina, NEB, Lpswich, MA, USA), then the library was initially quantified by Qubit2.0 Fluorometer. After the library is qualified, the different libraries are pooled according to the effective concentration and the target amount of data of the machine, then sequenced by the Illumina NovaSeq 6000. After quality control, differential expression analysis was performed using the DESeq2 R package (1.20.0). Heatmaps were drawn using the R package (Pretty Heatmaps; R package version 3.8.1). Mapping of gene expression levels to corresponding KEGG pathways was performed using the clusterProfiler R package (3.8.1).

### 2.7. Quantitative Real-Time PCR (RT-qPCR) Analysis

To validate the reliability of the RNA-seq results, a set of differentially expressed genes were chosen for RT-qPCR analysis. Total RNA was extracted as mentioned above. RNA was reverse transcribed using a PrimeScript TM RT reagent Kit with gDNA Eraser (RR047A, TaKaRa Bio Inc., Otsu, Shiga, Japan), and qPCR was performed using TB Green PCR Master Mix (RR820A, Takara Bio Inc., Otsu, Shiga, Japan) on ABI 7500 thermocycler (Applied Biosystems, CA, USA). Relative mRNA expression was calculated from the comparative threshold cycle (Ct) values. PCR primers were designed using online primer tools Primer3Plus and the sequences are provided in Table S1.

### 2.8. Statistical Analysis

All data are presented as the means ± SDs or median and interquartile range and were analyzed using one-way analysis of variance (ANOVA) with Tukey’s post hoc analyses or the Kruskal–Wallis test. Statistical analyses were accomplished using Prism version 8.0 (GraphPad Software Inc., San Diego, CA, USA) with *p* < 0.05 considered statistically significant.

## 3. Results

### 3.1. Metformin and Pioglitazone Combination Ameliorated HFD-Induced Metabolic Disturbance and NAFLD

**Figure 1:**
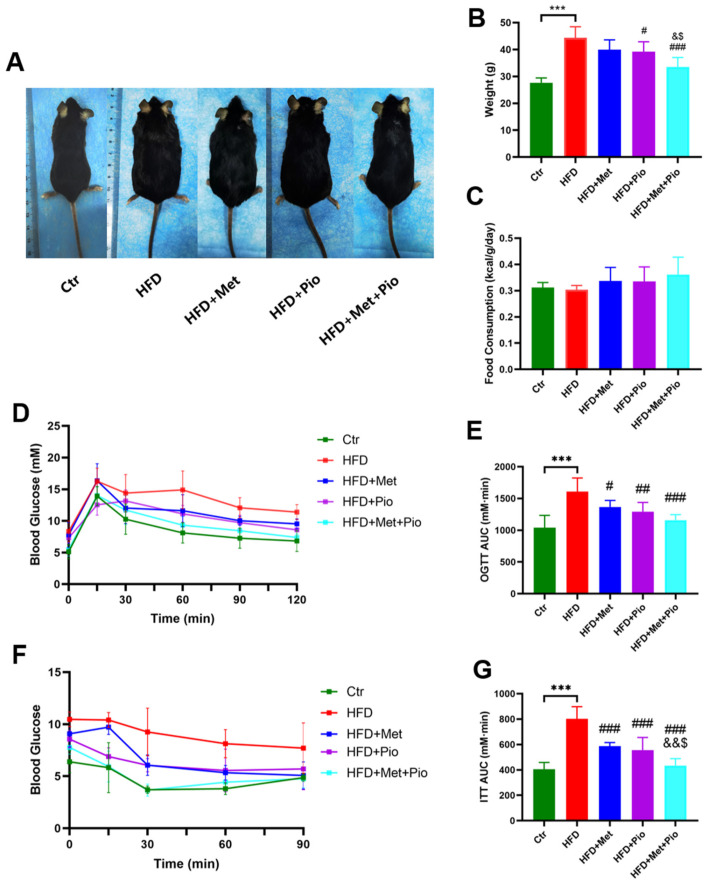
Metabolic phenotype ameliorated with combination therapy of metformin and pioglitazone. (**A**) General picture of body weight; (**B**) Body weight; (**C**) Food consumption; (**D**,**E**) Oral glucose tolerance test and the area under the curve; (**F**,**G**) Insulin tolerance test and the area under the curve. Ctr, standard control diet; HFD, high-fat diet; HFD + Met, high-fat diet treated with metformin; HFD + Pio, high-fat diet treated with pioglitazone; HFD + Met + Pio, high-fat diet treated with metformin and pioglitazone; OGTT, oral glucose tolerance test; ITT, insulin tolerance test; AUC, area under the curve. Data are expressed as the means ± SDs. (n = 6/group). One-way ANOVA; *** *p* < 0.001 HFD versus Ctr group; ^#^ *p* < 0.05, ^##^ *p* < 0.01, ^###^ *p* < 0.001 versus HFD group; ^&^ *p* < 0.05, ^&&^ *p* < 0.01 versus HFD + Met group; ^$^ *p* < 0.05 versus HFD + Pio group.

**Figure 2:**
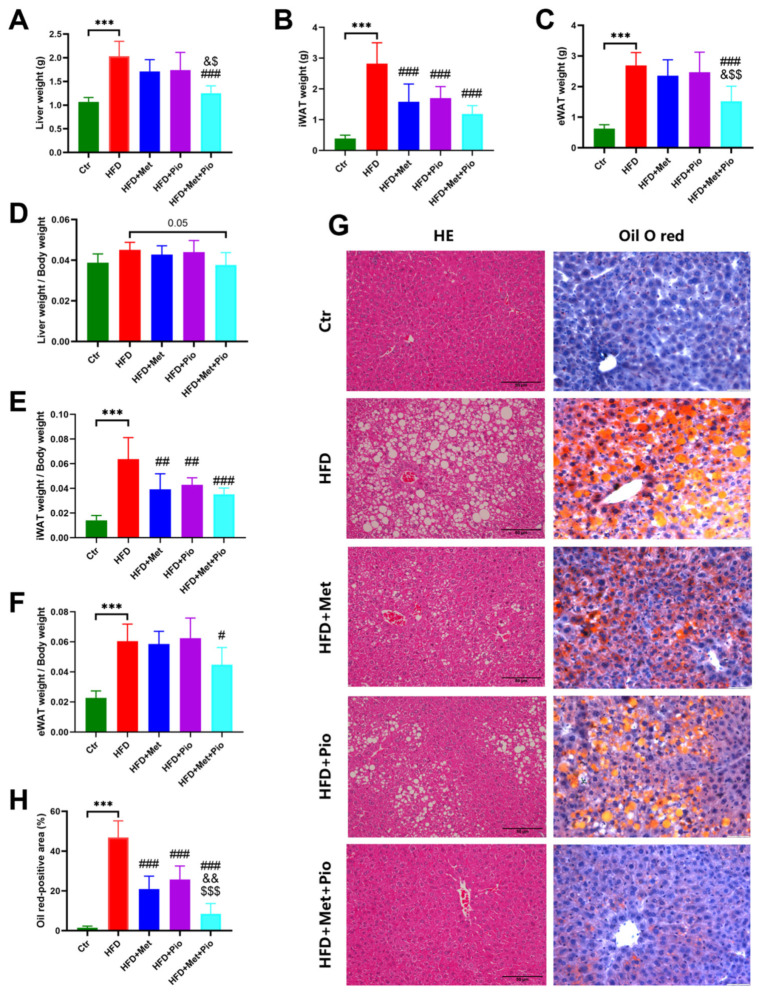
NAFLD phenotype ameliorated with combined therapy. (**A**) Liver weight; (**B**) iWAT weight; (**C**) eWAT weight; (**D**) Ratios of liver weight; (**E**) Ratios of iWAT weight; (**F**) Ratios of eWAT weight; (**G**) HE and oil O red staining; (**H**) Oil O red-positive area. Ctr, standard control diet; HFD, high-fat diet; HFD + Met, high-fat diet treated with metformin; HFD + Pio, high-fat diet treated with pioglitazone; HFD + Met + Pio, high-fat diet treated with metformin and pioglitazone; iWAT, inguinal white adipose tissue; eWAT, epididymal white adipose tissue; HE, hematoxylin and eosin; Data are expressed as the means ± SDs. (n = 6/group). One-way ANOVA; *** *p* < 0.001 HFD versus Ctr group; ^#^ *p <* 0.05, ^##^ *p* < 0.01, ^###^ *p* < 0.001 versus HFD group; ^&^ *p <* 0.05, ^&&^ *p* < 0.01 versus HFD + Met group; ^$^ *p <* 0.05, ^$$^ *p* < 0.01, ^$$$^*p* < 0.001 versus HFD + Pio group.

As shown in Figure 1 and Table 1, an HFD significantly induced obesity and the IR phenotype of the mice after a total of 18 weeks of HFD treatment, in line with previous results [24]. As expected, eight weeks of combined treatment with metformin and pioglitazone (HFD + Met + Pio group) had synergistic effects or at least additive effects on body weight reduction and ITT improvement compared with monotherapy intervention (*p* < 0.05). Fasting serum lipid levels were also significantly altered in the combined treatment group. The combination of metformin and pioglitazone significantly suppressed liver weight and was more effective than the monotherapy intervention (Figure 2A). Meanwhile, the weight of the white adipose tissues was also decreased in the intervention groups (Figure 2B,C). Liver and white adipose tissues presented as ratios relative to body weight are shown in Figure 2D–F. HE and oil red O staining showed that the combined treatment group had less hepatic oil red O area and significantly ameliorates NAFLD (Figure 2G,H). In addition, as listed in Table 1, the combined treatment group had significantly decreased levels of ALT.

### 3.2. Metformin and Pioglitazone Combination Significantly Altered Liver Lipid Profile in HFD Mice

**Figure 3:**
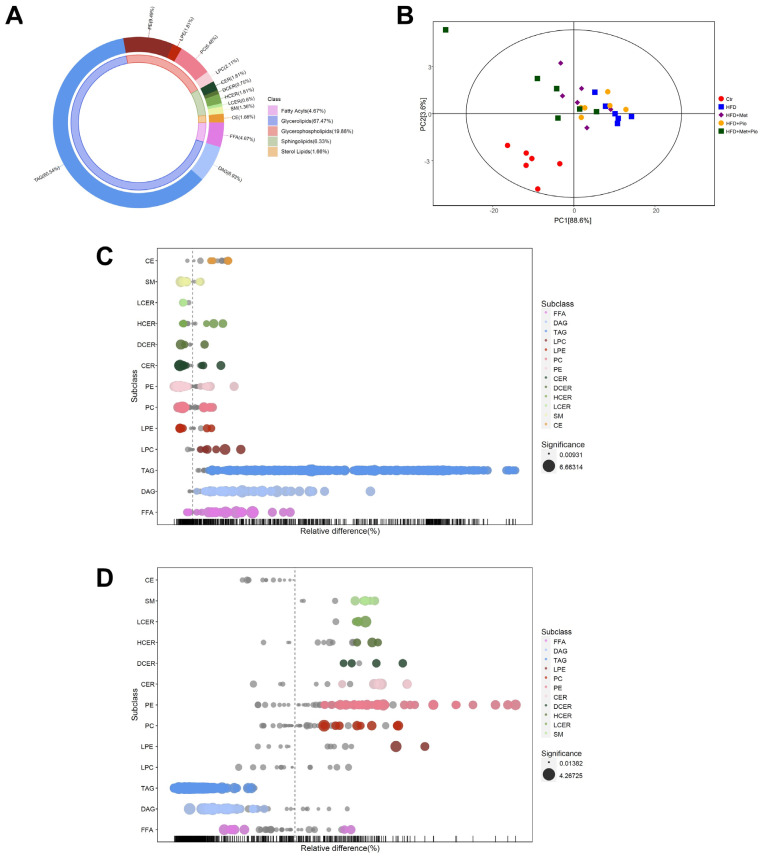
Lipid profiles altered in the combined treatment. (**A**) Donut chart of lipid subclass; (**B**) Principal component analysis; (**C**) Bubble plot in abundance of lipid species in HFD vs. Ctr; (**D**) Bubble plot in abundance of lipid species in HFD + Met + Pio vs. HFD. Ctr, standard control diet; HFD, high-fat diet; HFD + Met, high-fat diet treated with metformin; HFD + Pio, high-fat diet treated with pioglitazone; HFD + Met + Pio, high-fat diet treated with metformin and pioglitazone; CE, cholesterol ester; SM, sphingomyelin; LCER, lactosyl-ceramides; HCER, hexosyl-ceramides; DCER, dihydroceramides; CER, ceramides; PE, phosphatidyl ethanolamine; PC, phosphatidylcholine; LPE, lysophosphatidylethanolamine; LPC, lysophosphatidylcholine; TAG, triglyceride; DAG, diacylglycerol; FFA, free fatty acids.

To characterize the lipid profile in the livers of mice with metformin and pioglitazone intervention, we performed lipidomics analysis via LC-MS analysis. Lipidomics detected 664 lipids in total, including glycerolipids, glycerophospholipids, sphingolipids, fatty acyls and sterol lipids, which belong to 13 distinct subclasses (Figure 3A). The quality of the lipidomics data was evaluated using the consistency of quality control samples (Figure S1A). PCA analysis showed that the HFD group had a distinct lipid profile compared to that of the normal controls (Figure 3B). Further score scatter plots of orthogonal projections to latent structures-discriminant analysis models showed distinct separations between the HFD + Met group, HFD + Pio group and HFD + Met + Pio group vs. the HFD group (Figure S1B–D).

The relative differences in lipids were exhibited as a bubble plot on the basis of subclasses. As shown in Figure 3C and Table S2, 413 upregulated and 34 downregulated lipids were discovered between the Ctr and HFD groups. The alteration of lipid profiles by metformin and pioglitazone monotherapy intervention was mild (Figure S2A,B). Surprisingly, the metformin and pioglitazone combination almost reverse all the lipid subclasses changed by HFD consumption, indicating a significant lipid profile alteration by combination therapy (Figure 3D). A total of 454 differential lipids (42 upregulated and 412 downregulated) were between the HFD and combined therapy groups. Venn diagrams revealed that 394 altered lipids were shared by the HFD group vs. the Ctr group and the combined therapy group vs. the HFD group (Figure S2C). As shown in Figure 3C,D and Table S2, FFA, diacylglycerol (DAG) and TAG levels were significantly induced in the HFD group and reversed in the combined therapy group, while the number of glycerophospholipids and sphingolipids changed in the opposite direction.

### 3.3. Combination Therapy Regulated Gene Expressions in Liver

**Figure 4:**
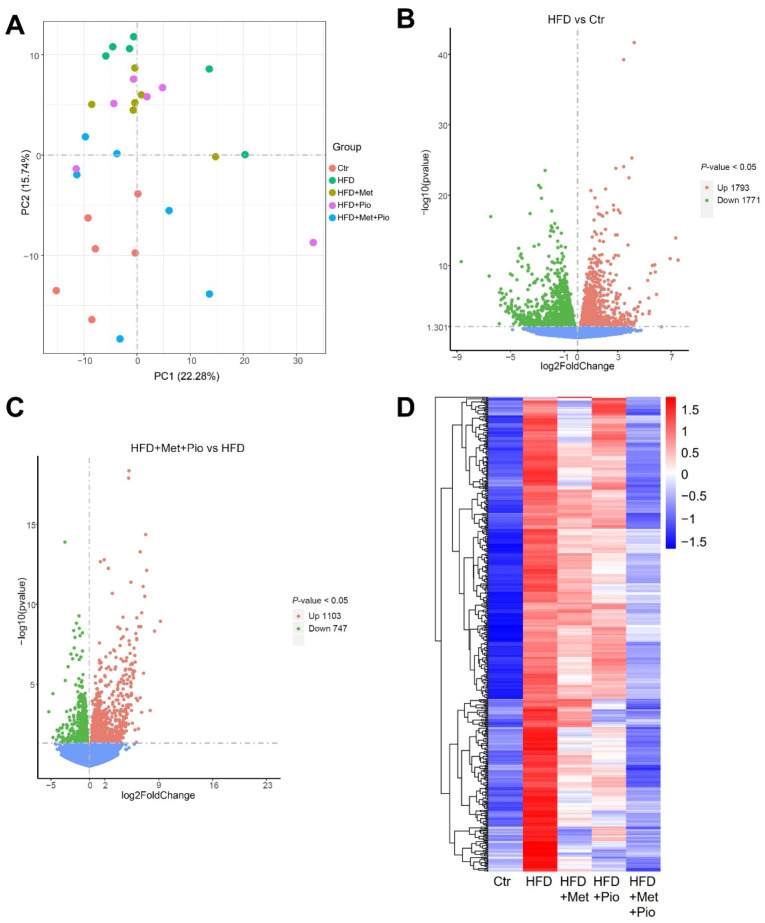
Gene expressed altered in the combined treatment. (**A**) Principal component analysis; (**B**) Volcano plot of gene expressed altered between HFD and Ctr; (**C**) Volcano plot of gene expressed altered between HFD + Met + Pio and HFD; (**D**) Heatmap of genes increased in HFD (vs. Ctr) and reduced in HFD + Met + Pio (vs. HFD) group. Ctr, standard control diet; HFD, high-fat diet; HFD + Met, high-fat diet treated with metformin; HFD + Pio, high-fat diet treated with pioglitazone; HFD + Met + Pio, high-fat diet treated with metformin and pioglitazone.

To investigate the genes that regulate lipid metabolism and reveal the mechanism of metformin/pioglitazone combination therapy on NAFLD, we performed an RNA-seq analysis. A total of 96.6 GB of clean data were obtained from 30 liver samples from the five groups (n = 6 per group). After read mapping to the reference genome, 54,532 genes were identified grossly. The Pearson R^2^ values between all samples in the five groups were over 0.8, suggesting the good repeatability of the samples. As shown in Figure 4A, the combined therapy group had a distinct separation from the HFD group and was nearest to the Ctr group in PCA.

A volcano plot illustrating the differentially expressed genes between the HFD and Ctr is shown in Figure 4C (1793 upregulated and 1771 downregulated genes) according to the *p*-value. A similar volcano plot between the combined therapy group and the HFD group is shown in Figure 4C. A total of 1850 genes were differentially expressed (1103 upregulated and 747 downregulated) in the combined therapy group (*p* < 0.05). Figure S3A,B shows the differentially expressed genes between the two monotherapy groups and the HFD group. We further explore the shared list of genes between HFD vs. Ctr and HFD + Met + Pio vs. HFD in Table S3. The heatmaps shown in Figure 4D and Figure S3C visualize the significantly different genes with two expression patterns. One type of gene expression was upregulated in the HFD (vs Ctr) and downregulated after HFD + Met + Pio treatment (Figure 4D). The other type of gene expression showed the opposite expression trends. In conclusion, from the RNA-seq data, we found that metformin and pioglitazone combined therapy had a significant reversal effect on hepatic gene expression induced by HFD consumption. However, the reversal effect was mild for either the metformin or pioglitazone monotherapy.

### 3.4. Functional Analysis of Altered Genes Regulated by HFD and Reversed by Combination Therapy

To explore the potential molecular pathway of the combined therapy effects on NAFLD, we used the clusterProfiler R package to test the statistical enrichment of differentially expressed genes in KEGG pathways.

**Figure 5:**
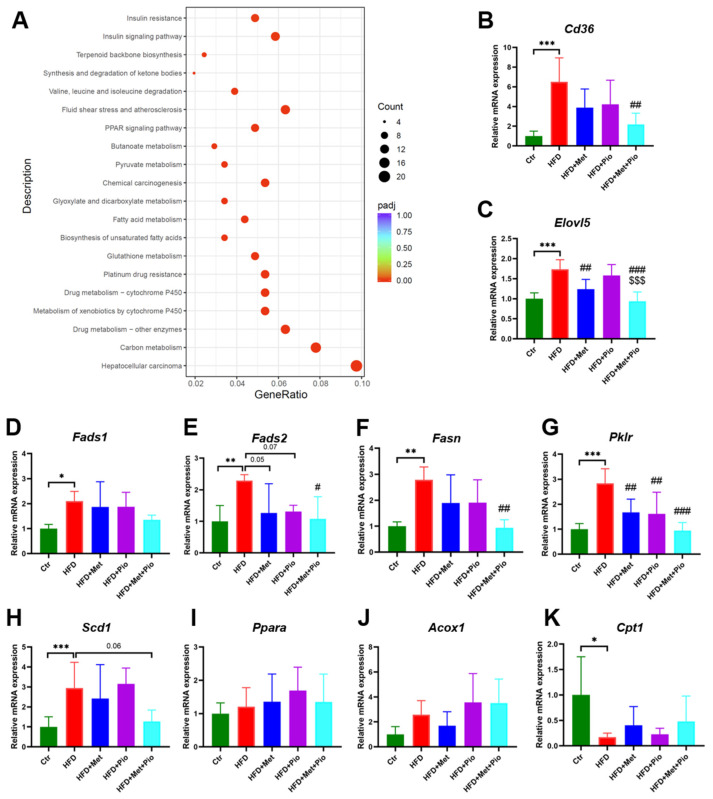
KEGG pathway enrichment and RT-qPCR validation of the differentially expressed genes. (**A**) KEGG pathway enrichment; (**B**–**K**) RT-qPCR validation of a set of genes in liver. Data are expressed as the means ± SDs. (n = 6/group). One-way ANOVA; * *p <* 0.05, ** *p* < 0.01, *** *p* < 0.001 HFD versus Ctr group; ^#^ *p <* 0.05, ^##^ *p* < 0.01, ^###^ *p* < 0.001 versus HFD group; ^$$$^ *p* < 0.001 versus HFD + Pio group.

We first analyzed the genes that were upregulated in the HFD group but substantially downregulated in the combined therapy group as demonstrated in Figure 4D. As shown in Figure 5A, these genes were mainly enriched in the signaling pathways associated with lipid metabolism, including biosynthesis of the unsaturated fatty acids, fatty acid metabolism, pyruvate metabolism, the PPAR signaling pathway and the insulin resistance pathway. In the biosynthesis of the unsaturated fatty acids pathway, *Fads1, Fads2, Elovl5, Scd1* and *Acot4* were significantly changed. Fatty acid synthase *Fasn*, which is an important lipogenic gene, was significantly changed in fatty acid metabolism. Among the pyruvate metabolism pathway, *Pklr* and *Acat2* were significantly changed. In addition, the related fatty acids metabolism is also enriched in the PPAR signaling pathway or insulin resistance pathway, such as *Fads2*, *Pparg*, *Acsl5*, *Fabp1* and *Acacb*. *Cd36*, participating in the uptake of FFA in the hepatocytes, is also induced in HFD and had a trend of reduction in the combined therapy group. Further validation experiments of part of these genes were conducted using RT-qPCR (Figure 5B–H).

In addition to a high affinity and potency for the PPARγ isoform, TZDs also showed a weak affinity for the α-subtype PPAR [27]. Previous studies have reported that PPARα target genes were involved in the fatty acid metabolism in tissues with high oxidative rates such as the liver [28]. In this study, we also verified the gene expressions of *Ppara* and two fatty acid β-oxidation activity-associated genes, *Acox1* and *Cpt1*. As shown in Figure 5I-K, *Ppara* and *Acox1* showed an increased trend in the pioglitazone monotherapy group and combined treatment group. However, there is no statistically significant difference. Relative gene expression of *Cpt1* was significantly decreased in the Ctr group, but pioglitazone intervention or combined treatment could not significantly reverse its expression.

We also discovered that the genes that were downregulated in the HFD group but substantially upregulated in the combined therapy group were mainly related to cysteine and methionine metabolism, complement and coagulation cascades, biosynthesis of amino acids and NOD−like receptor signaling pathway (Figure S3D).

**Figure 6:**
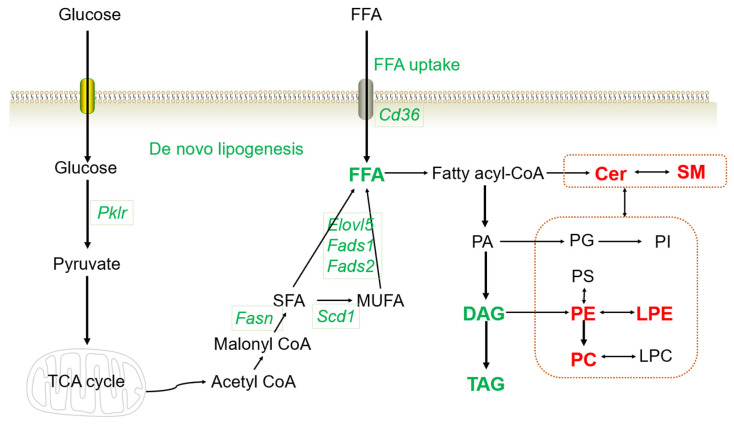
Altered lipid profiles and gene expression with the combined treatment. The genes or lipid metabolites in red color indicate upregulation, and green indicates downregulation in the combined therapy group. TCA cycle, tricarboxylic acid cycle; SFA, saturated fatty acid; MUFA, monounsaturated fatty acid; SM, sphingomyelin; Cer, ceramides; PE, phosphatidyl ethanolamine; PC, phosphatidylcholine; LPE, lysophosphatidylethanolamine; LPC, lysophosphatidylcholine; PA, phosphatidic acid; PI, phosphatidyl inositol; PS, phosphatidylserine; TAG, triglyceride; DAG, diacylglycerol; FFA, free fatty acids.

Combined with the RNA-seq and lipidomics results, the complicated disturbance in the hepatic lipid profiles induced by HFD consumption might be successfully reversed using a combination treatment of metformin and pioglitazone (Figure 6).

## 4. Discussion

NAFLD is characterized by the presence of more than 5% of hepatocytes with steatosis and is closely related to obesity and T2DM [2]. Previous evidence has suggested that pioglitazone and metformin therapy could ameliorate NAFLD [4,8,13,14,15,16]. However, the combined effects and molecular mechanisms of pioglitazone and metformin on NAFLD have not yet been illustrated. In our previous study, we found that pioglitazone and metformin combined therapy had synergistic effects, or at least an additive effect, on the gut microbiota in obese mice [24]. In the present study, we further investigated the protective effect of the combined therapy in HFD-induced NAFLD via lipidomics and RNA-seq analysis.

Liver steatosis is caused by dysregulation of lipid homeostasis and a disproportionate amount of the uptake, synthesis and utilization of lipids, leading to the accumulation of toxic lipids [29]. Consistently, in the present study, we confirmed that the HFD induced a NAFLD phenotype along with increased FFA, DAG and TAG levels. In line with previous research, we also confirmed that metformin and pioglitazone monotherapy could partly reverse the harmful variation tendency. Interestingly, the vast majority of FFA, DAG and TAG induced by HFD were reduced in content by the metformin and pioglitazone combined treatment group. At the same time, liver weight and lipid droplets, as assessed by oil red O staining, were significantly decreased. These data indicated that pioglitazone and metformin combined therapy significantly reversed HFD-induced NAFLD in C57BL/6 male mice.

There might be numerous mechanisms responsible for the reduced levels of FFA, DAG and TAG in the combined therapy group. We performed RNA-seq in liver tissue samples to explore the underlying pathways for the protective effects. Interestingly, we found that genes induced by HFD consumption and reversed in the combined group were mainly enriched in the signal pathways associated with lipids metabolism, including biosynthesis of unsaturated fatty acids, fatty acid metabolism, pyruvate metabolism, PPAR signaling pathway and insulin resistance pathway. The genes enriched in the biosynthesis of unsaturated fatty acids and fatty acid metabolism pathways included *Fads1, Fads2, Fasn, Scd1* and *Elovl5*. *Fads1* and *Fads2* belong to the fatty acid desaturase gene cluster, key regulators of polyunsaturated fatty acid biosynthesis [30]. *Fads1* overexpression increased FFA synthesis and TAG accumulation in goat mammary epithelial cells by inhibiting the AMP-activated protein kinase (AMPK) pathway [31], and the inhibition of *FADS1* reversed the arachidonic acid and TAG levels in the HepG2 cells under harmful cadmium ion exposure [32]. *Fasn* was verified to promote the generation of palmitate (FA 16:0) from acetyl-CoA [33], and palmitate was induced in the HFD and reversed by pioglitazone and metformin combined therapy consistently in our lipidomics results. A previous study found that hepatic *SCD1* expression increased in NAFLD patients and ob/ob mice, and berberine, a natural compound extracted from a Chinese herb, could alleviate NAFLD and reduce the expression of *Fasn* by promoting the AMPK pathway [26]. Metformin is an AMPK activating agent and pioglitazone mainly targets *PPARγ*, which regulates the transcription of a vast number of insulin and lipid metabolism-related genes. Therefore, it is reasonable to speculate that metformin and pioglitazone have synergistic effects for the reduced synthesis of FFA and TAG.

The pyruvate metabolism pathway was another important enriched pathway in our results. A set of genes in the pyruvate metabolism pathway were significantly increased in the HFD group and decreased in the combination intervention group, such as *Pklr*. Liver pyruvate kinase *Pklr*, a key enzyme in glycolysis, has been proven to be a regulator of hepatic lipid metabolism and mitochondrial function [34,35]. *Pklr* catalyzes the production of pyruvate, which can enter into the tricarboxylic acid cycle and accelerate the de novo lipogenesis [35]. Evidence has shown that *Pklr* knockout could reverse the increased hepatic TAG accumulation in mice with NAFLD and significantly improve the transcriptomic changes [35,36]. Therefore, *Pklr* might play a critical role during the progression of NAFLD and be a potential therapeutic target via de novo lipogenesis [35]. However, few studies have researched the effect of metformin or pioglitazone on hepatic *Pklr* expression in NAFLD. One study found that metformin could counteract the elevated expression of *Pklr* induced by high glucose in vitro [37]. In the present results, we found that metformin or pioglitazone monotherapy could partly reverse the high *Pklr* expression levels induced by the HFD and the combined therapy group strongly reduced *Pklr* levels, indicating that the underlying synergistic protective mechanism of the reduced FFA and TAG levels in the metformin and pioglitazone combined therapy group might be decreasing the glucose uptake and inhibiting de novo lipogenesis.

In addition to the reduced levels of FFA, DAG and TAG in the combined therapy group, a vast number of glycerophospholipids and sphingolipids increased. Glycerophospholipids and sphingolipids are both crucial elements of the cell membrane and have many signal transduction roles [38,39]. In our present study, the subclasses of significantly increased glycerophospholipids were a set of phosphatidyl ethanolamine (PE), phosphatidylcholine (PC) and lysophosphatidylethanolamine (LPE). Previous studies have reported that glycerophospholipids levels decreased in both HFD-fed mice and NAFLD patients, suggesting that dysregulation of glycerophospholipids might lead to the disturbance of membrane stability and cause the pathogenesis of hepatic steatosis [39,40]. Sphingolipids mainly consist of ceramides (Cer) and sphingomyelin (SM) and were considered lipotoxic in the liver in many previous studies [41]. Nevertheless, evidence could also be found that the alteration of specific lipid molecules in the SM−Cer pathway might lead to the progression of NAFLD [39]. Accumulating evidence has suggested a bidirectional homeostatic crosstalk between glycerophospholipids and sphingolipids and their imbalance might cause a series of metabolic disorders [42]. Although the underlying mechanism of the increased levels of glycerophospholipids and sphingolipids in the metformin and pioglitazone combined therapy group was unclear and still needs further investigation, evidence could be found that the PC and metformin attenuating lipopolysaccharide induced toll-like receptor 4 and pro-inflammatory cytokines overexpression depending on whether there is a combined presence of NAFLD [43].

In conclusion, the results of our present study demonstrated that combination therapy with metformin and pioglitazone has significant protective effects on NAFLD at both the phenotypic and molecular levels in the HFD-induced mice. It appears that the combined intervention group has a more efficient effect than the monotherapy group. Metformin and pioglitazone synergistically reduced ALT levels, liver weight, hepatic lipid droplets, FFA, DAG and TAG levels, along with decreased expression of genes associated with FFA uptake and de novo lipogenesis, such as *Fads1, Fads2, Fasn, Scd1, Elovl5, Pklr* and *Cd36.* Therefore, it is reasonable to suggest that the combination of metformin and pioglitazone might be a promising treatment strategy for NAFLD. However, the reversal effects on glycerophospholipids and sphingolipids and the complex underlying mechanism still need further exploration. Further studies are also needed to verify the clinical effects.

## Figures and Tables

**Table 1:** Serum biochemical parameters among different groups.

Parameters	Ctr	HFD	HFD + Met	HFD + Pio	HFD + Met + Pio
Glucose (mmol/L)	5.28 ± 2.05	11.51 ± 1.51 ***	10.31 ± 1.90	8.72 ± 1.27	8.58 ± 1.24 ^#^
Insulin (ng/mL)	0.25 (0.18–0.65)	1.13 (0.89–1.38) **	0.80 (0.71–1.18)	0.77 (0.35–1.17)	0.42 (0.31–0.68) ^##^
HOMA-IR	1.61 (0.81–3.01)	10.81 (9.38–17.04) **	7.86 (5.09–12.85)	6.02 (2.64–10.49)	3.56 (2.31–5.81) ^#^
TAG (mmol/L)	0.47 ± 0.09	0.41 ± 0.15	0.39 ± 0.16	0.37 ± 0.11	0.40 ± 0.08
TC (mmol/L)	2.79 ± 0.92	6.32 ± 0.34 ***	5.05 ± 1.38	5.00 ± 0.59 ^#^	3.36 ± 0.95 ^###^
LDL-C (mmo/L)	0.30 ± 0.07	0.61 ± 0.21 ***	0.43 ± 0.05	0.42 ± 0.09 ^#^	0.34 ± 0.10 ^##^
FFA (mmol/L)	950.67 ± 185.01	615.80 ± 54.39 ***	735.33 ± 85.63	708.07 ± 43.44	813.07 ± 157.40 ^#^
AST (u/L)	205.67 ± 45.26	228.60 ± 19.53	210.60 ± 24.73	197.20 ± 48.20	203.20 ± 37.42
ALT (u/L)	28.87 ± 6.89	60.3 ± 13.46 **	44.27 ± 18.10	51.87 ± 26.11	37.93 ± 12.47 ^##$^

HOMA-IR, homeostasis model assessment of insulin resistance; TAG, triglyceride; TC, total cholesterol; LDL-C, low-density lipoprotein cholesterol; FFA, free fatty acids; AST, aspartate transaminase; ALT, alanine aminotransferase. Data are expressed as the means ± SDs or median and interquartile range. (n = 6/group). One-way ANOVA with Tukey’s post hoc analyses or Kruskal–Wallis test; ** *p* < 0.01, *** *p* < 0.001 HFD versus Ctr group; ^#^ *p <* 0.05, ^##^ *p* < 0.01, ^###^ *p* < 0.001 HFD + Met group versus HFD group, HFD + Pio group versus HFD group or HFD + Met + Pio group versus HFD group; ^$^ *p <* 0.05 HFD + Met + Pio group versus HFD + Pio group.

## Data Availability

The datasets generated for this study can be found in the Sequence Read Archive (SRA) database (PRJNA999231).

## Supplementary Materials

The following supporting information can be downloaded at: https://www.mdpi.com/article/10.3390/biom13081199/s1, Table S1: list of primer sequences used for RT-PCR analysis in this study. Table S2: list of altered lipid species. Table S3: list of different expressed genes. Figure S1: PCA analysis and orthogonal projections to latent structures-discriminant analysis models of lipidomics analysis. Figure S2: lipid profiles altered in monotherapy and combined treatment. Figure S3: gene expressed altered in monotherapy and combined treatment.

## Abbreviations

NAFLD, nonalcoholic fatty liver disease; HFD, high-fat diet; FFA, free fatty acids; T2DM, type 2 diabetes mellitus; IR, insulin resistance; TZDs, thiazolidinediones; PPARγ, peroxisome proliferator-activated receptor γ; AMPK, AMP-activated protein kinase; OGTT, oral glucose tolerance test; ITT, insulin tolerance test; AUC, area under the curve; TAG, triglyceride; TC, total cholesterol; ALT, alanine aminotransferase; AST, aspartate aminotransferase; HOMA-IR, homeostasis model assessment of insulin resistance; HE, hematoxylin and eosin; LC-MS, liquid chromatography-mass spectrometry; PCA, principal component analysis; VIP, variable important in projection; RT-qPCR, quantitative real-time PCR; ANOVA, one-way analysis of variance; DAG, diacylglycerol; PE, phosphatidyl ethanolamine; PC, phosphatidylcholine; LPE, lysophosphatidylethanolamine; Cer, ceramides; SM, sphingomyelin.
