# Supplementary material for: Combination of Pioglitazone and Metformin Actions on Liver Lipid Metabolism in Obese Mice
Source: Biomolecules. 2023 Jul 31;13(8):1199. doi: 10.3390/biom13081199 (PMC10452643; doi:10.3390/biom13081199)

Supplementary Materials

Table S1. List of primer sequences used for RT-PCR analysis in this study.

Primer	Forward (5'to3')	Reward (5'to3')
Elovl5	GGTGGCTGTTCTTCCAGATT	CCCTTCAGGTGGTCTTTCC
Scd1	CCGGAGACCCCTTAGATCGA	TAGCCTGTAAAAGATTTCTGCAAACC
Fads1	TCAGTCTTTGGCACCTCGAC	TCCTTGCGGAAGCAGTTAGG
Fads2	GGACTTCGTGGGCAAGTTCT	CAGTGCCGAAGTACGAGAGG
Fasn	GCTGCGGAAACTTCAGGAAAT	AGAGACGTGTCACTCCTGGACTT
Pklr	CCCGAGATACGCACTGGAG	CGACCTGGGTGATATTGTGGT
Cd36	GGAGTGGTGATGTTTGTTGCT	GCACACACCACCATTTCTTCT
Gapdh	TGTGTCCGTCGTGGATCTGA	CCTGCTTCACCACCTTCTTGAT
Ppara	TACTGCCGTTTTCACAAGTGC	AGGTCGTGTTCACAGGTAAGA
Acox1	CCTGATTCAGCAAGGTACGG	TCGCAGACCCTGAAGAAATC
Cpt1	TCTAGGCAATGCCGTTCAC	GAGCACATGGGCACCATAC

Table S2. List of altered lipid species.
Uploaded as a separate file in Excel.
Ctr, standard control diet; HFD, high-fat diet; HFD+Met, high-fat diet treated with metformin; HFD+Pio, high-fat diet treated with pioglitazone; HFD+Met+Pio, high-fat diet treated with metformin and pioglitazone.

Table S3. List of different expressed genes.
Uploaded as a separate file in Excel.
Ctr, standard control diet; HFD, high-fat diet; HFD+Met+Pio, high-fat diet treated with metformin and pioglitazone.

Figure S1. PCA analysis and orthogonal projections to latent structures- discriminant analysis models of lipidomics analysis. (A) PCA analysis of lipidomics analysis with QC; (B) Orthogonal projections to latent structures-discriminant analysis models between HFD and HFD+Met group; (C) Orthogonal projections to latent structures-discriminant analysis models between HFD and HFD+Pio group; (D) Orthogonal projections to latent structures-discriminant analysis models between HFD and HFD+Met+Pio group. PCA, principal component analysis; Ctr, standard control diet; HFD, high-fat diet; HFD+Met, high-fat diet treated with metformin; HFD+Pio, high-fat diet treated with pioglitazone; HFD+Met+Pio, high-fat diet treated with metformin and pioglitazone; QC, quality control.

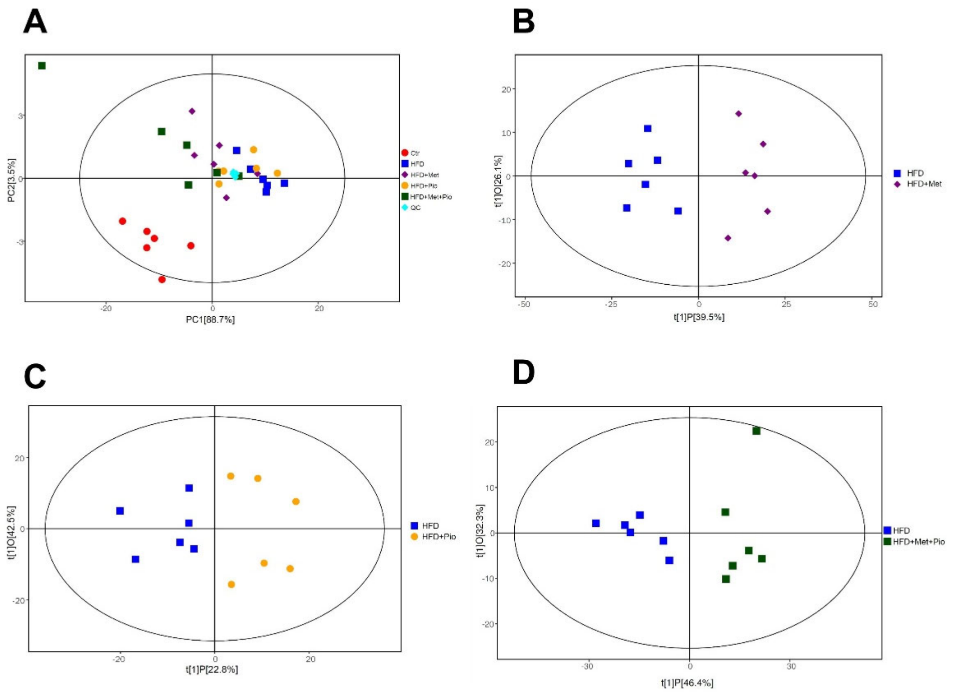

Figure S2. Lipid profiles altered in monotherapy and combined treatment.

(A) Bubble plot in abundance of lipid species in HFD + Met vs. HFD; (B) Bubble plot in abundance of lipid species in HFD + Pio vs. HFD; (C) Venn diagram of the shared lipid species changed in HFD + Met + Pio vs.HFD and HFD vs. Ctr. Ctr, standard control diet; HFD, high-fat diet; HFD + Met, high-fat diet treated with metformin; HFD + Pio, high-fat diet treated with pioglitazone; HFD + Met + Pio, high-fat diet treated with metformin and pioglitazone.

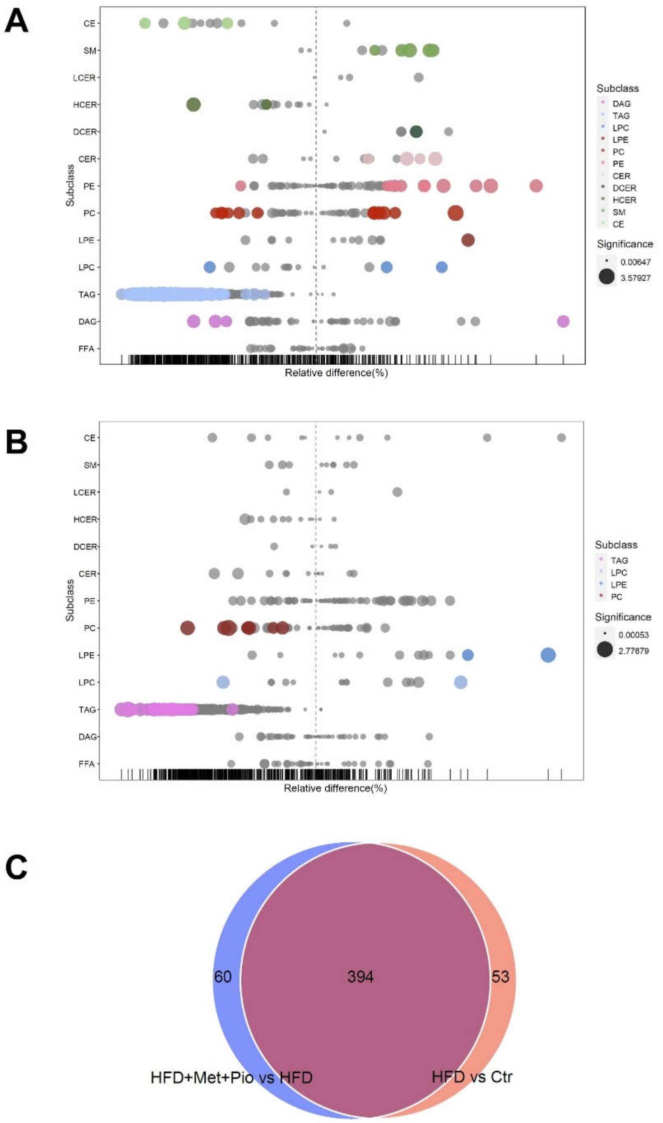

Figure S3. Gene expressed altered in monotherapy and combined treatment.

(A) Volcano plot of gene expressed altered between HFD + Met and HFD; (B) Volcano plot of gene expressed altered between HFD + Pio and HFD; (C) Heatmap of genes decreased in HFD (vs. Ctr) and increased in HFD + Met + Pio (vs. hfd) group; (D) KEGG pathway enrichment of the differentially expressed genes. Ctr, standard control diet; HFD, high-fat diet; HFD + Met, high-fat diet treated with metformin; HFD + Pio, high-fat diet treated with pioglitazone; HFD + Met + Pio, high-fat diet treated with metformin and pioglitazone.

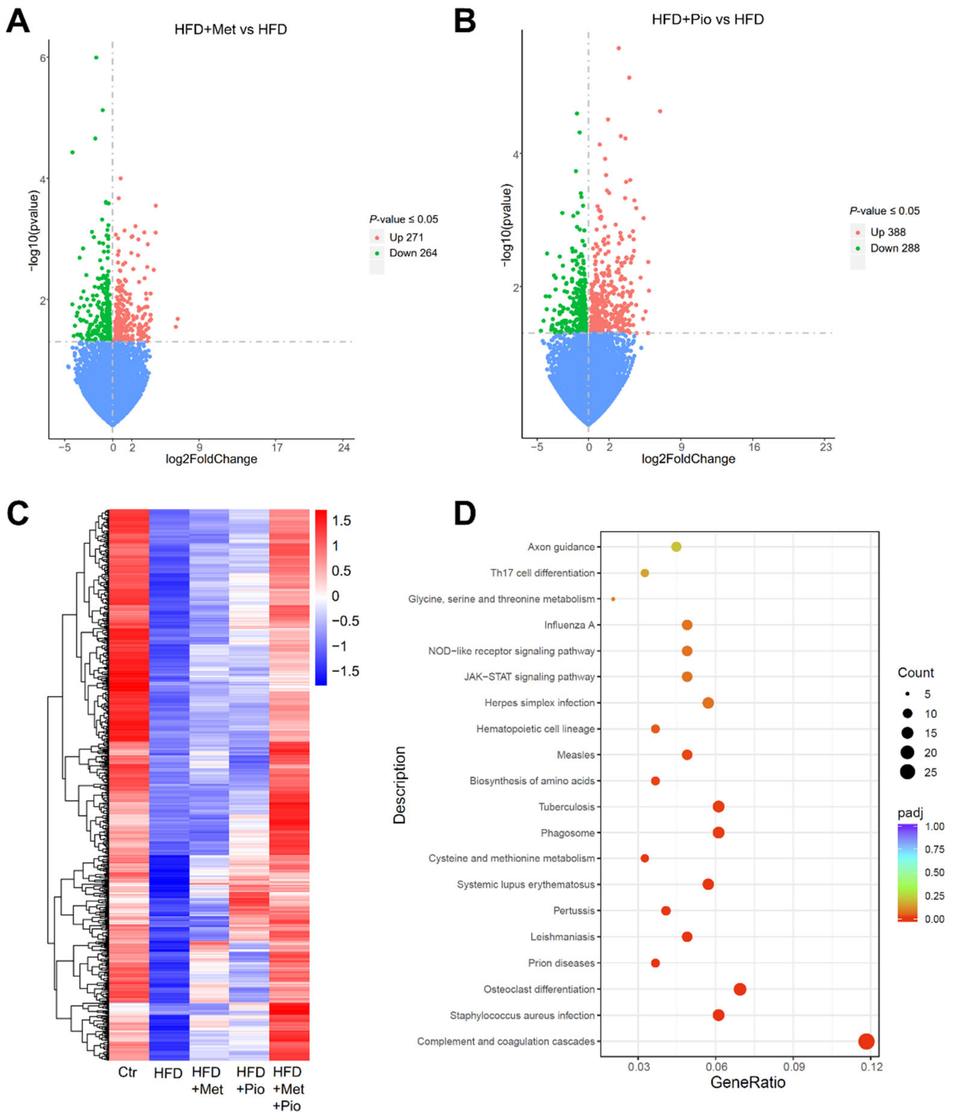

Supplement: Supplementary file 1 [file biomolecules-13-01199-s001.zip › Supplementary materials.pdf]
